# Beneficial Impacts of Incorporating the Non-Natural Amino Acid Azulenyl-Alanine into the Trp-Rich Antimicrobial Peptide buCATHL4B

**DOI:** 10.3390/biom11030421

**Published:** 2021-03-12

**Authors:** Areetha R. D’Souza, Matthew R. Necelis, Alona Kulesha, Gregory A. Caputo, Olga V. Makhlynets

**Affiliations:** 1 Department of Chemistry, Syracuse University, 111 College Place, Syracuse, NY 13244, USA; ardsouza@syr.edu (A.R.D.); akulesha@syr.edu (A.K.); 2 Department of Chemistry & Biochemistry, Rowan University, Glassboro, NJ 08028, USA; neceli48@students.rowan.edu (M.R.N.); caputo@rowan.edu (G.A.C.); 3 Department of Molecular & Cellular Biosciences, Rowan University, Glassboro, NJ 08028, USA

**Keywords:** antimicrobial peptides, fluorescence, lipid binding, buCATHL4B, non-natural amino acids, protease resistance, membrane permeabilization

## Abstract

Antimicrobial peptides (AMPs) present a promising scaffold for the development of potent antimicrobial agents. Substitution of tryptophan by non-natural amino acid Azulenyl-Alanine (AzAla) would allow studying the mechanism of action of AMPs by using unique properties of this amino acid, such as ability to be excited separately from tryptophan in a multi-Trp AMPs and environmental insensitivity. In this work, we investigate the effect of Trp→AzAla substitution in antimicrobial peptide buCATHL4B (contains three Trp side chains). We found that antimicrobial and bactericidal activity of the original peptide was preserved, while cytocompatibility with human cells and proteolytic stability was improved. We envision that AzAla will find applications as a tool for studies of the mechanism of action of AMPs. In addition, incorporation of this non-natural amino acid into AMP sequences could enhance their application properties.

## 1. Introduction

Antimicrobial peptides (AMPs) have been an area of intensive and multidisciplinary investigation through the last three decades. The interest in these peptides is driven by multiple factors including the small size, high selectivity, low cytotoxicity, and broad evolutionary presence in higher organisms. Importantly, these AMPs have also been of great interest due to the potential pharmaceutical applications noting the duration of these molecules throughout evolution, indicating a low potential for resistance development. More recently, novel applications beyond the traditional pharmaceutical approach have begun to develop using AMPs as components of hydrogels and as antimicrobial surface coatings [1].

A major area of interest in AMPs is the design and development of novel molecules with high activity and bioavailability but with low toxicity to the host. In this pursuit, numerous groups have studied AMPs from a variety of sources to gain insights into the structure–activity relationships within these peptides [2,3,4,5]. However, despite this sustained effort over the past 30 years, there has been no consensus sequence identified or reliable prediction methods for the design of new AMPs. Generally, the majority of these peptides are found to be cationic and amphiphilic, containing a significant number of hydrophobic amino acids, and act via a membrane-disrupting mechanism [6,7]. This membrane disruption is driven by the peptide adopting a facially amphiphilic alpha-helix on the membrane surface which allows the hydrophobic amino acids to insert into the core of the lipid membrane, causing destabilization.

Equally important in the development and application of novel AMPs as therapeutics is the relationship between selectivity, efficacy, and host cytocompatibility. Efficacy has long been a hurdle for AMPs due to concerns of bioavailability, primarily stemming from the natural host mechanisms for digesting proteins. One strategy that many groups have been using to approach the bioavailability problem is the incorporation of non-natural amino acids which may be less susceptible to these proteolytic mechanisms. While this has shown success in a number of cases, the overall understanding of the physico-chemical properties of these amino acids is more limited compared to natural amino acids. Perhaps the most important challenge is developing AMPs with low cytotoxicity to host cells. While AMPs evolved to be part of the immune response in higher organisms, and are thus biocompatible, the modification of these molecules has the potential to disrupt the evolved balance of efficacy and cytocompatibility. Importantly, it has been shown that increasing the hydrophobicity of AMPs and AMP-mimetics can dramatically increase toxicity [8,9,10,11]. These changes in toxicity are likely linked to changes in binding and pore-forming propensity as a result of the modulation of the hydrophobic balance in the peptide as has been demonstrated for the AMP Magainin H2 [12,13,14]. Thus, any changes in the AMP sequences must be carefully vetted to ensure any increases in antibacterial activity are not concomitant with increases in broad toxicity.

**Scheme 1:**
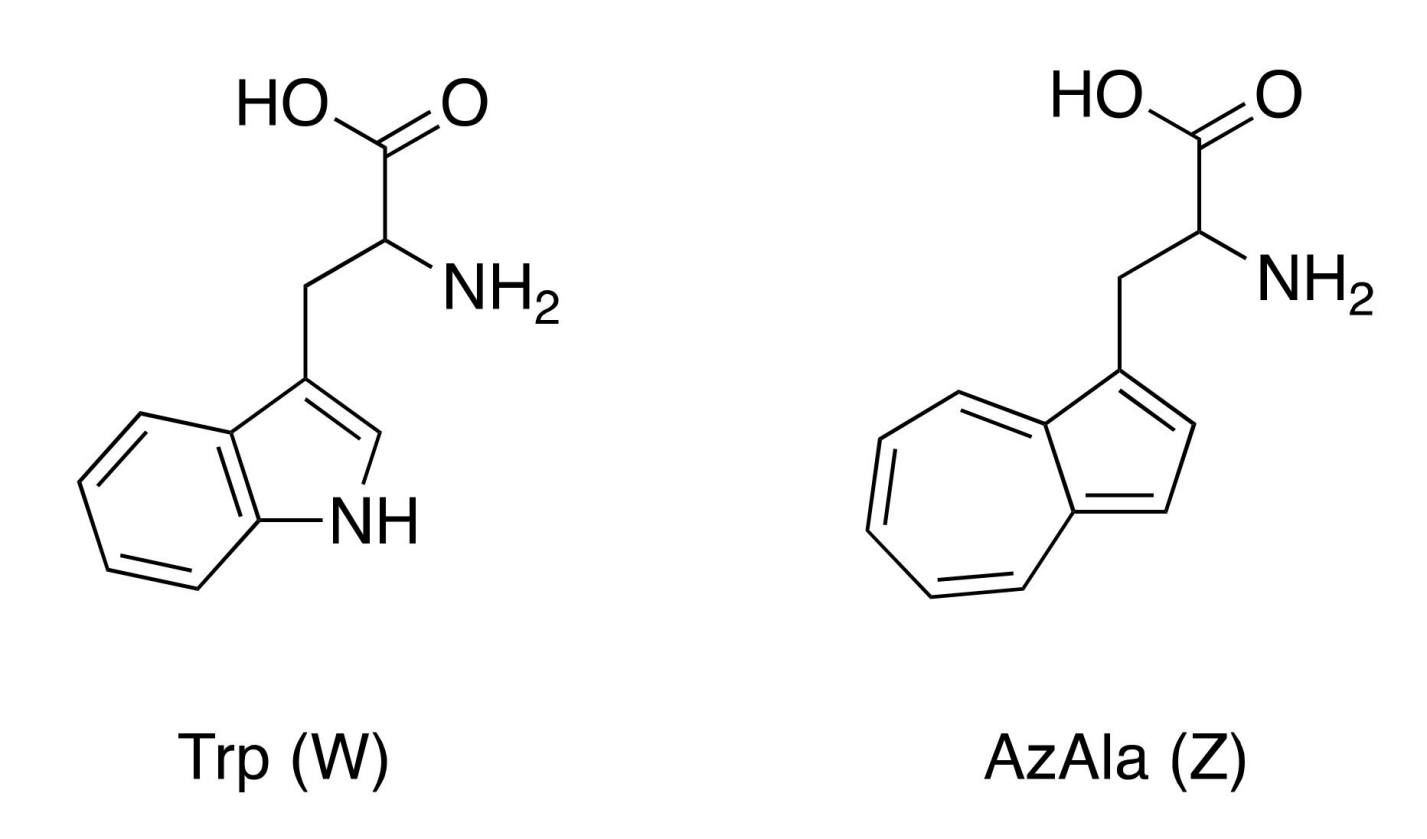
Structures of tryptophan (Trp, W) and its analog Azulenyl-Alanine (AzAla, Z).

More recently, AMPs that diverge from the traditional cationic amphiphilic model have been identified including anionic AMPs and Trp-rich AMPs (tryptophan = Trp). The Trp-rich class of AMPs has garnered significant interest as these molecules following the general cationic-amphiphilic model of most AMPs, but with several significant differences. First, as per the nomenclature, these peptides have multiple Trp residues in their sequences, well above the statistically predicted number for peptides of this length. The Trp-rich class of AMPs is generally much shorter in length than typical AMPs, often only 10–20 amino acids long. In order to study the mechanism of action of AMPs, fluorescence provides for an easy readout that can be monitored in a high-throughput fashion in various environments (detergent micelles, lipid bilayers, and even in vivo). The challenge of incorporating a fluorophore into a target peptide lies in balancing response with retention of natural function. Extrinsic labeling with relatively large fluorophores engineered into a protein may perturb its structure and function. Intrinsic fluorescent probes for such studies are of great value, as they are potentially less disruptive to the structure of the biological object of interest. The ability to be excited separately from all other residues, high sensitivity to the local environment, and relatively low abundance in proteins make tryptophan particularly well suited for such studies [15]. Moreover, the sensitivity of tryptophan to the environment [16] and locally present quenchers offers useful information about probe localization [17,18]. However, some of Trp’s advantages can also be weaknesses in other scenarios [19]. The sensitivity of Trp to both the local environment and quenchers inherently present in proteins (or other biomolecules) can convolute the analysis of the fluorescence data. For example, when several events (such as binding, quenching, and drastic change of local environment) occur simultaneously and lead to concomitant change in ϕ (quantum yield) and λ_max_ (emission maximum). Additionally, sequence modifications are necessary for peptides that contain multiple intrinsic fluorophores in order to get information for a distinct site. An approach to address this problem is to substitute the residue of interest with a non-natural analog, which can be selectively excited above ~310 nm, where Trp absorption is negligible. Ideally, such substitution should only minimally perturb native peptide’s properties. This could be achieved by developing iso- or pseudoisosteric mimics with distinct fluorescence properties [15,20,21,22]. We identified azulene (Az), a pseudoisosteric hydrocarbon analog of indole that lacks the N-H functionality (Scheme 1), which can serve as an environmentally insensitive probe for fluorescence studies of AMPs while preserving (and even improving) their functionality. Azulene has a number of advantages: its absorption spectrum is well separated from all other amino acid residues, the fluorescence emission photophysics is simple, the quantum yield value for azulene is comparable to that of tryptophan, and it does not possess functional groups that render it sensitive to the local environment [23]. Previously, it was demonstrated that the incorporation of AzAla into the well-characterized venom peptide melittin did not impact the structure/function of the peptide [24]. However, melittin is a longer, membrane-spanning peptide which forms distinct pores in the bilayer, in contrast to the general consensus for AMPs. In this work, we synthetically incorporated azulene into Trp-containing AMP to demonstrate that AzAla does not perturb peptide function and could serve as a probe to dissect the roles and importance of individual Trp residues in multi-Trp AMPs.

Using a combination of spectroscopic, microbiological, and biochemical assays, we have characterized the AzAla-containing variants of buCATHL-4B. Most notably, while retaining antimicrobial activity, all of these variants show reduced cytotoxicity to mammalian red blood cells and mouse fibroblasts.

## 2. Materials and Methods

### 2.1. Materials

Isopropyl β-D-1-thiogalactopyranoside (IPTG) (Chem-Impex Int’l., Wood Dale, IL, USA), ortho-Nitrophenyl-β-galactoside (ONPG) (Research Products International Co., Mt Prospect, IL, USA), and nitrocefin (Biovision, Milpitas, CA, USA) were stored as powders at −20 °C and prepared fresh for each use. Fmoc-protected amino acids were purchased from GL Biochem Shanghai Ltd. China and Rink Amide MBHA resin from Chem-Impex. Trypsin powder was obtained from Amresco (Solon, OH, USA). Solid POPC lipid (>99% purity) was purchased from Avanti Polar Lipids (Alabaster, AL, USA). Non-natural amino acid Fmoc-β-(1-Azulenyl)-L-Alanine was synthesized according to previously published protocols [23,25].

### 2.2. Peptide Synthesis and Purification

The peptides were synthesized by manual Fmoc solid-phase synthesis at elevated temperature using Rink Amide MBHA resin and Fmoc-protected amino acids using previously reported protocols [26]. Final peptides were uncapped at the N-terminus and contained C-terminal amide group. Coupling of Fmoc-β-(1-Azulenyl)-L-Alanine was performed for 30 min at room temperature. Cleavage of the peptides from the resin and side-chain deprotection were simultaneously achieved by treatment with a mixture of trifluoroacetic acid (TFA)/H_2_O/triisopropyl silane (TIS) (95:2.5:2.5, *v*/*v*) for 2 h at room temperature. The crude peptides were precipitated and washed with cold methyl-tert-butyl ether and purified on a Shimadzu (Kyoto, Japan) preparative reverse phase High-Performance Liquid Chromatography system with a Jupiter C4 preparative column (Phenomenex, Torrance, CA USA), using a linear gradient of solvent A (0.1% TFA in MilliQ water) and solvent B (90% CH_3_CN, 9.9% MilliQ water, 0.1% TFA). A gradient of 35–65% of solvent B was used at a flow rate of 20 mL/min for 30 min to purify the peptides. The identities of the peptides were confirmed using a Bruker (Billerica, MA USA) MALDI-TOF mass spectrometer. Purity of the obtained peptides was evaluated on an Agilent Infinity II 1260 (Santa Clara, CA USA) with an analytical Zorbax Eclipse XDB-C18 column by Agilent (4.6 mm × 150 mm). Peptide stock solutions in water were prepared from the lyophilized powder (<90% purity). Lyophilized peptides are stable at −20 ˚C for three months, and solutions of peptides need to be prepared immediately before the experiment.

### 2.3. Trypsin Digestion

Pure lyophilized buCATHL4B (WWW) peptides were dissolved in MilliQ water (obtained using Elix Millipore, Burlington, MA USA) and syringe filtered (0.2 micron, 6500 rpm for 10 min) prior to checking concentration. The concentration of WWW peptide was determined by measuring absorbance at 280 nm on the UV–Vis spectrophotometer (Agilent 8453, Agilent Technologies, CA, USA) using ɛ_280_= 16,500 M^−1^cm^−1^ (Expasy). The concentrations of AzAla variants were determined by measuring absorbance at 342 nm on the UV–Vis spectrophotometer using ɛ_342_= 4212 M^−1^cm^−1^ [27]. The peptide stocks of 2 mM concentration were prepared in buffer (50 mM sodium phosphate, 100 mM NaCl, pH 7.0). Trypsin (1 mg/mL) stock was prepared fresh in buffer and serial dilutions were done for the assay: 0.1 mg/mL, 0.01 mg/mL, 0.001 mg/mL. The reaction samples (500 µL final volume) were prepared in triplicate by mixing WWW (500 µM), trypsin (50 µL of 0.001 mg/mL solution) in buffer (50 mM sodium phosphate, 100 mM NaCl, pH 7.0). The trypsin digest was monitored on the HPLC (Shimadzu) every 15 min at room temperature by following the peak of undigested peptide. Identity of various peaks in HPLC chromatogram were identified by MALDI-TOF. For this, fractions containing peptides corresponding to different peaks on HPLC were collected, lyophilized, and redissolved in 10 µL of solvent B (90% acetonitrile, 9.9% MilliQ water and 0.1% TFA); 10 µL of solvent A (99.9% MilliQ water and 0.1% TFA), and then 2 µL of CHCA (α-Cyano-4-hydroxycinnamic acid) matrix was added (1:10 proportion) and the mixture was loaded onto the MALDI target.

### 2.4. Circular Dichroism Spectroscopy

The circular dichroism (CD) spectra were acquired on the Jasco J-715 CD spectrometer (Easton, MD USA) collecting 64 scans (4 s averaging time) for each spectrum and using a quartz cuvette with a 1 cm path length. The measurements were performed on samples containing peptides (5 µM) in buffer (5 mM phosphate, 10 mM NaCl, pH 7.0) in the presence and in the absence of vesicles (250 µM of 100% POPC). Care was taken that the sample absorbance never exceeded 1.5 at all wavelengths to produce reliable ellipticity values. Mean residue ellipticity (MRE; deg × cm^2^ × dmol^−1^) values were calculated using the following equation, where *θ* is ellipticity (mdeg), *l* is pathlength (cm), *C* is peptide concentration (M), and *N* is number of residues.

(1) $$\mathrm{MRE}\;=\;\frac{\theta }{10*C*l*N}$$

### 2.5. Fluorescence

Fluorescence data for the peptides were obtained on a JY-Horiba 914D fluoromax-2 spectrofluorometer (Horiba Scientific, NJ USA) at room temperature. Emission spectra measurements were taken in Spectrosil quartz cuvettes (Starna Cells, Atascadero, CA USA; 1 cm path length cuvette, sample volume 2 mL) using a 5 mm excitation slit width and 5 mm emission slit width for spectra. The measurements were performed on samples containing peptides (2 µM) in buffer (50 mM sodium phosphate, 150 mM NaCl, pH 7.0). The excitation wavelength used to excite AzAla was 342 nm. Fluorescence emission spectra were recorded over the range of 355 nm to 455 nm. To excite tryptophan, the excitation wavelength used was 280 nm. Fluorescence emission spectra were recorded over the range of 300 to 400 nm.

### 2.6. Preparation of Peptide in Vesicles

Solid POPC (12.5 mg) was dissolved in chloroform (0.5 mL) to make a lipid stock (16.5 mM). This stock (303 µL) was pipetted into a glass vial and dried with nitrogen gas and then under vacuum for 1 h. Then, ethanol (10 µL of 100%) was added to the film and vortexed until the film was completely dissolved. This was further resuspended in PBS buffer (5 mM sodium phosphate, 10 mM NaCl, pH 7.0) containing peptide (final concentration = 2 µM).

### 2.7. Minimal Inhibitory Concentration and Minimal Bactericidal Concentration Analysis

Bacteria were streaked from frozen glycerol stocks onto LB agar plates (*E. coli* D31, *S. aureus* ATCC 35556, *P. aeruginosa* PA-01, *A. baumannii* ATCC 19609). The plates were incubated at 37 °C for ~18 h to allow growth. An individual colony from each plate was transferred into individual sterile tubes containing 3 mL of Muller Hinton (MH) broth and subsequently incubated for 18 h with shaking (225 rpm) at 37 °C. An aliquot of this culture was then diluted with fresh MH broth (1:250) and further incubated with shaking (225 rpm) at 37 °C until an OD_600_ of 0.2–0.4 was reached. This mid-log culture was then diluted to 5 × 10^5^ CFU/mL in fresh MH broth. Serially diluted peptides were dispensed in a 96-well plate such that the final concentration in each well would be in the range of 15 µM to 0.234 µM. Then, 90 µL of the freshly diluted culture was transferred to the 96-well plate, yielding a final volume of 100 µL. The 96-well plate was covered and transferred to a humidified incubator at 37 °C for 18 h. The growth and inhibition of bacterial growth were determined by measuring OD_600_ directly in the plate and comparing to an untreated control. The Minimum Bactericidal Concentration (MBC) was determined by transferring 1 µL of culture from each well of the MIC plate onto fresh LB agar plates and allowed to incubate overnight at 37 °C. MBC is determined by the corresponding concentration from the MIC plate that resulted in no colony growth.

### 2.8. E. coli Outer Membrane Permeability

An individual colony of *E. coli* D31 was transferred into a sterile tube containing 3 mL of Luria Bertani (LB) broth supplemented with 100 µg/mL of ampicillin and subsequently incubated for 18 h with shaking (225 rpm) at 37 °C. An aliquot of this culture was then diluted with fresh LB broth (1:250) again supplemented with 100 µg/mL of ampicillin to induce the expression of β-lactamase and further incubated for with shaking (225 rpm) at 37 °C until the culture reached an OD_600_ of 0.2–0.4. The culture was then centrifuged at 2500 rpm for 15 min in a benchtop clinical centrifuge (Clay Adams), the supernatant was discarded, and the pellet was resuspended in an equal volume of PBS (100 mM sodium phosphate, 200 mM NaCl, pH 7). Peptides were serially diluted in 0.01% acetic acid in the same concentration ranges as used in the MIC experiments. A series of the antimicrobial peptide polymyxin B sulfate was included as a positive control. In a clear, flat bottom 96-well plate, 10 µL of the serially diluted peptide was added to each well followed by 80 µL of the resuspended *E.coli* culture, and 10 µL of 5 mg/mL nitrocefin substrate (dissolved in PBS). Immediately upon the addition of the substrate, the samples were briefly mixed by pipetting and absorbance at 486 nm was measured every 5 min for total of 90 min. Data reported is the average of 3 replicates.

### 2.9. E. coli Inner Membrane Permeability

An individual colony of *E. coli* D31 was transferred into a sterile tube containing 3 mL of Luria Bertani (LB) broth and subsequently incubated for 18 h with shaking (225 rpm) at 37 °C. An aliquot of this culture was then diluted with fresh LB broth (1:250) supplemented with 100 µL of 100 mM IPTG to induce the expression of β-galactosidase and further incubated for with shaking (225 rpm) at 37 °C until the culture reached an OD_600_ of 0.2–0.4. Peptides were serially diluted in 0.01% acetic acid in the same concentration ranges as used in the MIC experiments. A series of the detergent cetyl-trimethyl ammonium bromide (CTAB) was included as a positive control. In a clear, flat bottom 96-well plate, 10 µL of the serially diluted peptide was added to each well followed by 56 µL Z-buffer (60 mM Na_2_HPO_4_, 40 mM NaH_2_PO_4_, 10 mM KCl, 1mM MgSO_4_, 50 mM β-mercaptoethanol, pH 7), 19 µL of the *E.coli* culture, and 15 µL of 4 mg/mL ONPG substrate (dissolved in Z-buffer). Immediately upon the addition of the ONPG, the samples were briefly mixed by pipetting and absorbance at 420 nm was measured every 5 min for total of 90 min. Data reported is the average of 3 replicates.

### 2.10. Hemolysis

Fresh defibrinated blood from sheep (Hardy Diagnostics, Santa Maria, CA, USA) was transferred to a sterile centrifuge tube and diluted 10-fold with cold, sterile PBS for a total of 15 mL. The sample was centrifuged for 6 min in a clinical benchtop centrifuge. The supernatant was removed, and the pellet was gently resuspended to 15 mL with PBS. This was repeated for a total of 3 washes. After the 3rd centrifugation, the pellet was resuspended in PBS and 90 μL of the red blood cells (RBCs) were transferred to each well of a 96-well round-bottom plate containing 10 μL serially diluted peptides for a final volume of 100 μL in the wells. Wells containing PBS or Triton X-100 were used as the negative and positive controls, respectively. The plate was incubated at 37 °C for 60 min and subsequently centrifuged for 5 min to pellet remaining intact RBCs. Next, 6 µL of the supernatant from each well was transferred to a new 96-well plate containing 94 µL of PBS in each well and the absorbance of each well was measured at 409 nm and 415 nm. Percent hemolysis was calculated by normalizing the values from the negative and positive control wells to 0% and 100% hemolysis, respectively. All experiments were performed at least in triplicate. Error bars represent the standard deviations.

### 2.11. Cytocompatibility of buCATHL4B Peptide and its Derivatives with 3T3 Cells

Pure lyophilized peptides were prepared in 25% ethanol as 10X stocks and spin-filtered at 6500 rpm for 10 min (0.22 µm nylon membrane centrifugal filter, VWR) prior to checking concentration. The concentrations of WWW and AzAla-substituted peptides were determined as described in the Section 2.3.

Mouse embryonic fibroblast cells (3T3, ATCC) were a kind gift from Dr. Mary Beth Monroe’s lab (BioInspired Institute, Syracuse University). The cells were cultured in the complete medium consisting of Dulbecco’s Modified Eagle’s Medium (DMEM) with 4.5 g/L glucose and sodium pyruvate (Corning, Manassas, VA USA), supplemented with 2 mM L-glutamine (Gibco, Gaithersburg, MD USA), 10% fetal bovine serum (Gibco) and 1% penicillin–streptomycin mix (Gibco) at 37 °C, 5% CO_2_ and in high humidity environment. When cell monolayer reached 70–80% confluency, the cells were detached after incubation with trypsin–EDTA mixture (Gibco) and the cell quantity was determined using hemocytometer with trypan blue (Gibco) staining to estimate the number of live cells.

After 10,000 cells per well in 100 µL were seeded into black 96-well plates with clear bottom (Greiner Bio-One, Monroe, NC USA), the plates were incubated at 37 °C, 5% CO_2_ (high humidity) for 20–24 h. For the experiment, 80 µL of fresh complete medium and 20 µL of 10× peptide stock (the final concentration of ethanol in the culture was 2.5%) were added, and the plates were incubated for 5 h under cell culture conditions. Resazurin assay was performed to evaluate cell viability. Resazurin was added to the final concentration of 0.67 mM and fluorescence of resorufin, the product of resazurin reduction by living cells, was measured after 4 h of incubation using Biotek (Winooski, VT USA) Synergy 2 plate reader (excitation at 530 nm, emission at 590 nm). Cells without peptide treatment (25% ethanol was added instead of the peptide stock) were used as a positive control and cells treated with 3% H_2_O_2_ (Fisher) were used as a negative control. Percent viability was calculated as (F_peptide treated samples_ − F_negative control_)/ (F_positive control_ − F_negative control_) × 100% for an average of 3 runs.

## 3. Results

### 3.1. Peptides

**Figure 1:**
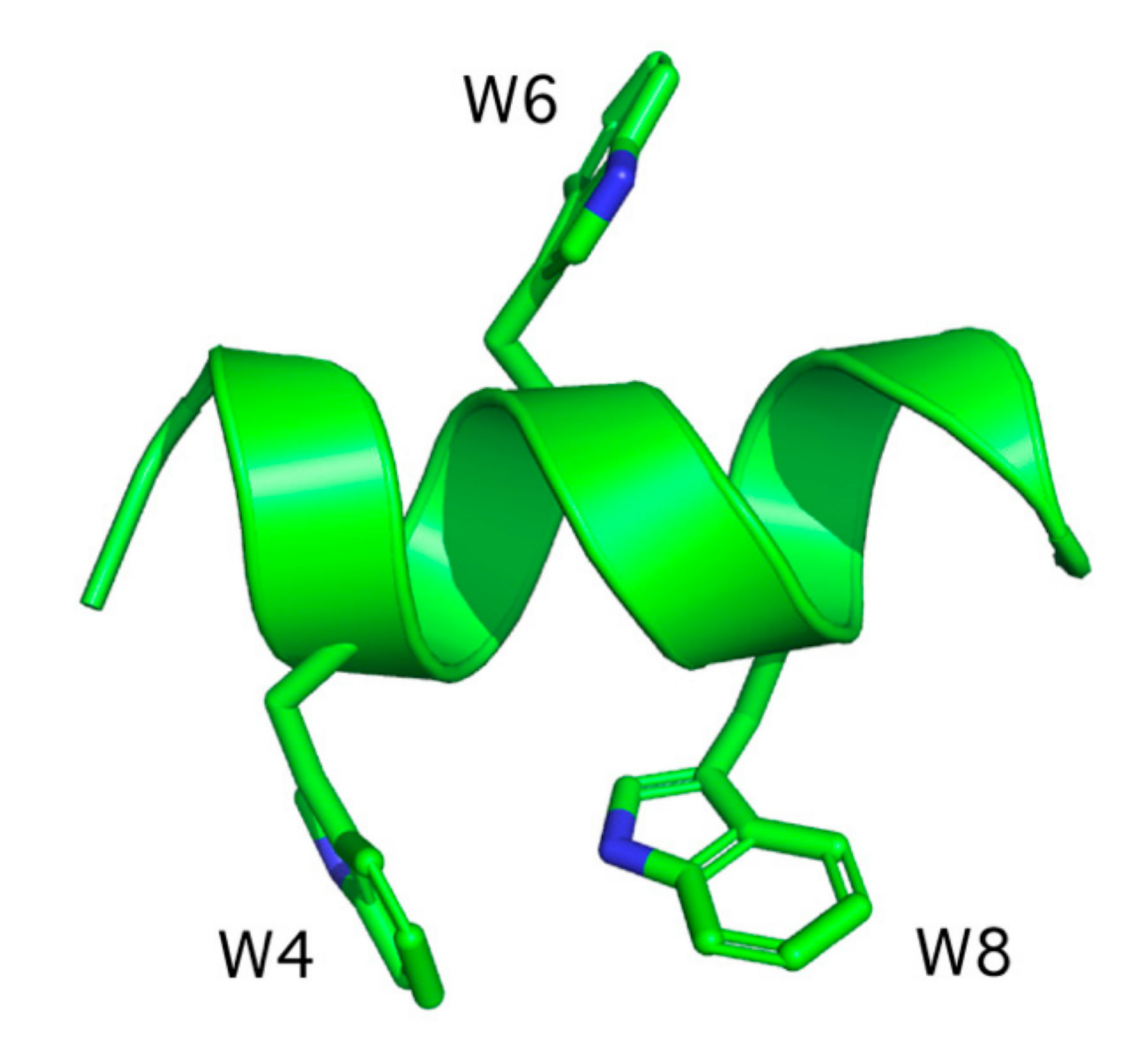
Peptide WWW with three tryptophan residues (W) marked as W4, W6, and W8. The model was generated using Phyre2 tool and then visualized in PyMOL.

To test the individual effects of Trp to AzAla substitutions, we designed three variants of buCATHL-4B in which each of the Trp residues was replaced with the non-natural amino acid β-(1-azulenyl)-L-alanine (AzAla, Z, Table 1) [23]. BuCATHL-4B, an antimicrobial peptide identified from genomic sequencing of five different breeds of Asian water buffalo (*Bubalis bubalis*), is a part of a 12-member family of Trp-rich peptides linked to cathelicidin gene 4 (of 7). Based on Phyre2 simulations (Figure 1), peptide is expected to adopt alpha-helical structure [28].

### 3.2. Antimicrobial Activity

The original study on buCATHL-4B confirmed peptide’s antimicrobial activity against both Gram-positive and Gram-negative bacteria [29]. The peptide increased bacterial membrane permeability and at low concentrations was shown to enhance the expression of several proinflammatory cytokines [29]. The antimicrobial activity of the peptides against Gram-positive *S. aureus* and Gram-negative *E. coli, P. aeruginosa*, and *A. baumannii* was determined using a traditional broth microdilution assay. The results are shown in Table 2 and compared to the previously reported activity of the parent peptide (WWW) [30]. While MIC determines the lowest concentration of peptide required to inhibit bacterial growth, it does not inform on the mechanism of this process. Thus, minimal bactericidal concentration (MBC) was determined by plating treated bacterial cultures on antibiotic-free solid media (Table 2, Supplemental Figure S1). The MBC results closely tracked with the MIC values, indicating the peptides are acting in a bactericidal manner and not by bacterial growth arrest. These results indicate that the substitution of Trp with AzAla has minimal, if any, effect on antimicrobial activity, with the possible exception of Z8 which exhibited a 4-fold decrease in MBC for Gram-negative *S. aureus* compared to the wild type peptide.

### 3.3. Bacterial Membrane Permeabilization

**Figure 2:**
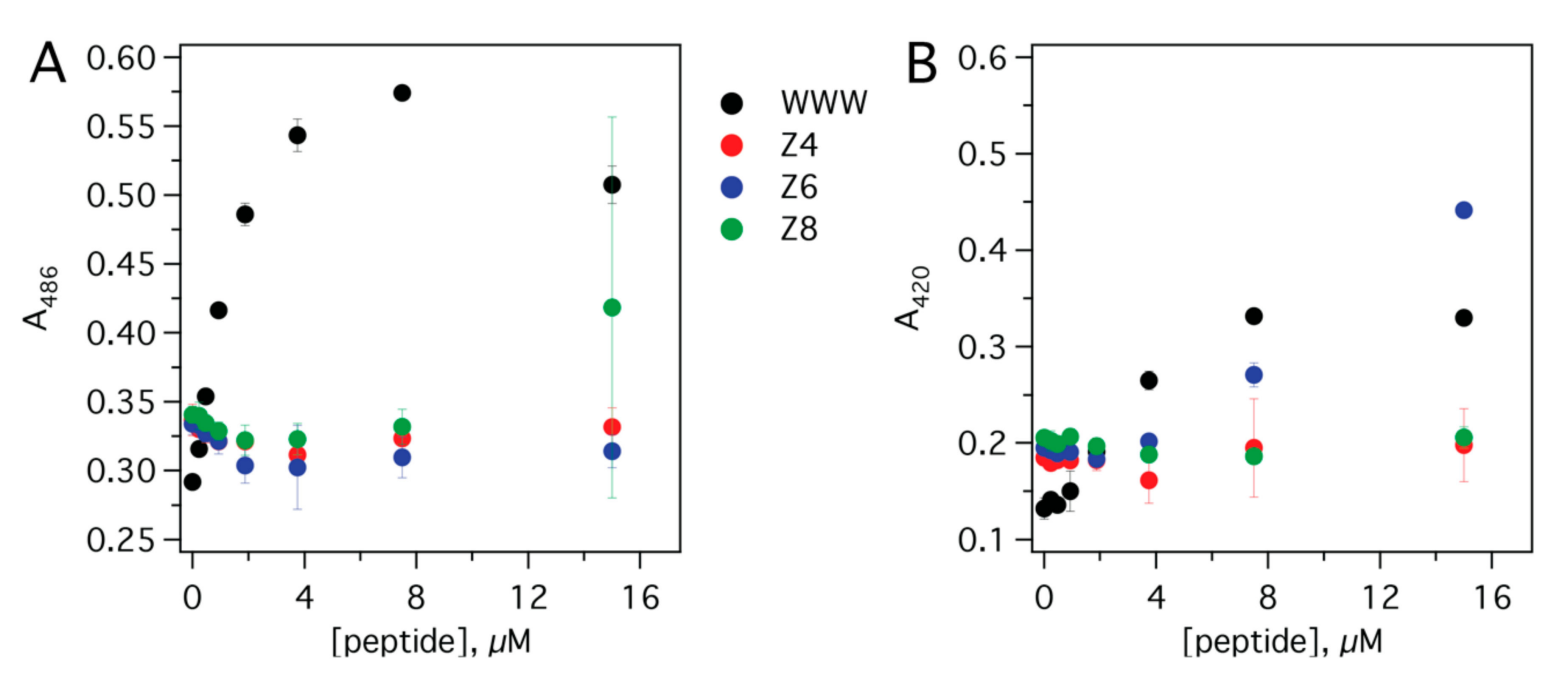
Bacterial membrane permeabilization. The permeabilization of the bacterial (**A**) outer membrane and (**B**) inner membrane is shown after 30 min of exposure to peptides. The chromogenic substrates used are (**A**) nitrocefin and (**B**) ONPG. Data shown are the averages of 3 trials with standard deviations.

The widely accepted mechanism of action for many AMPs is the permeabilization of the bacterial membrane. To investigate the influence of substitutions on peptide’s activity, the AzAla-containing peptides were screened for the ability to disrupt the *E. coli* outer and inner membranes [31,32]. Briefly, membrane impermeable chromogenic substrates were used to assay for compartment-specific enzymes in the periplasmic space (β-lactamase and substrate nitrocefin, to assay outer membrane permeability) and the cytoplasm (β-galactosidase and substrate ONPG, to assay inner membrane permeability) [33,34]. An increase in membrane permeability allows enhanced transport of the substrates across the membrane allowing enzymatic hydrolysis, resulting in a chromophore detectable by absorbance spectroscopy. Interestingly, none of the AzAla-substituted peptides caused any outer membrane permeabilization, as opposed to the parent peptide (Figure 2A, Figures S2 and S3). In contrast, while the parent, Z4, and Z8 peptides induced no permeabilization of the inner membrane, the Z6 peptide caused some delayed damage beginning after 20–30 min of exposure to the highest test concentrations (15 µM and 7.5 μM).

### 3.4. Circular Dichroism to Determine Peptide Structural Effects

**Figure 3:**
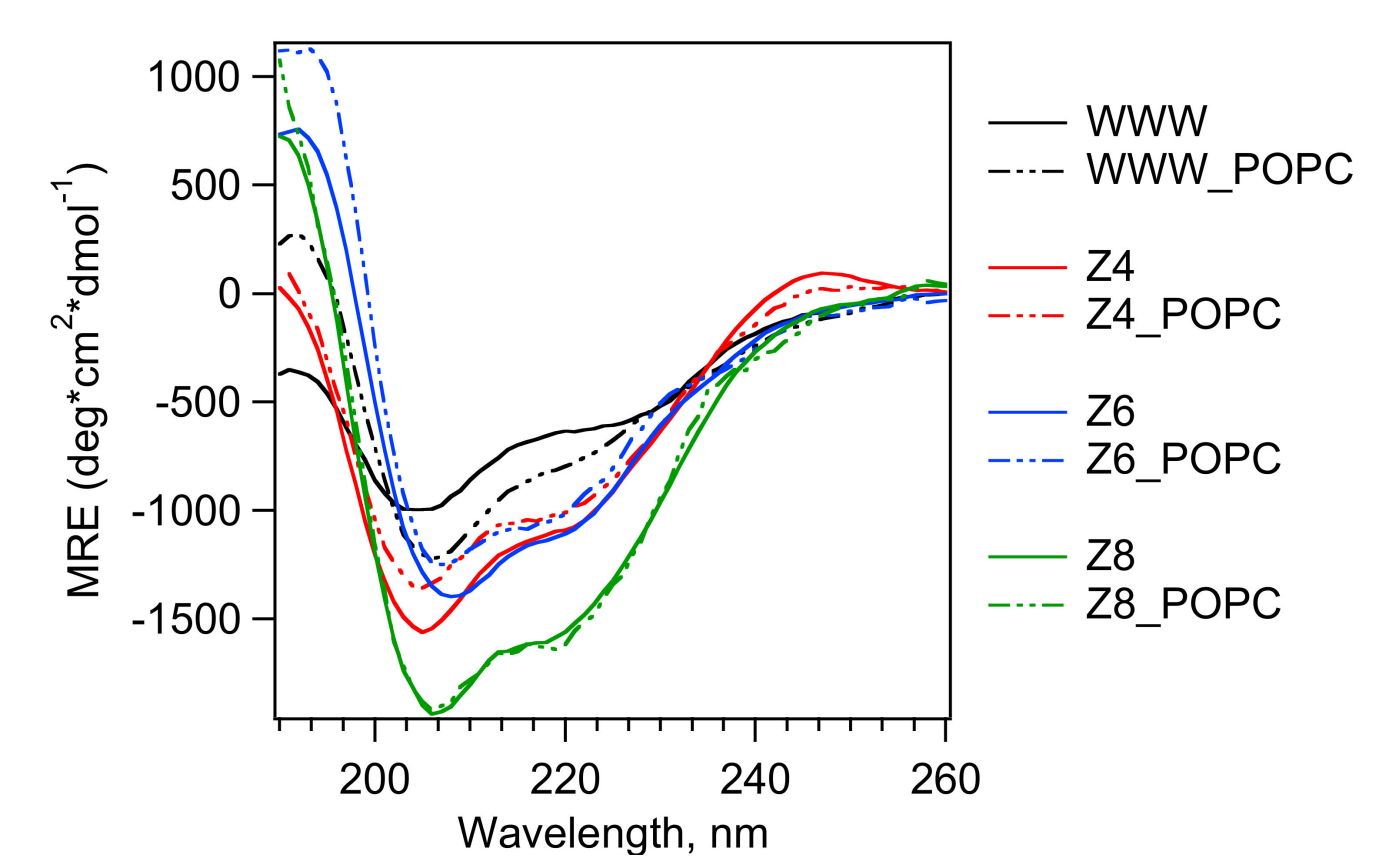
Circular dichroism (CD) spectra of peptides without (solid line) and with POPC lipid (dotted line). Conditions: [peptides] = 5 µM, [POPC] = 250 µM, buffer = 5 mM phosphate, 10 mM NaCl, pH 7.0.

The proposed mechanism of action for AMPs often involves the formation of an α-helical secondary structure upon binding to the bacterial membrane surface [35,36,37]. This structural transition is important for helical AMPs as it results in the formation of a facially amphiphilic structure with hydrophobic amino acids sequestered to one face of the helix, promoting membrane insertion. Circular dichroism (CD) can be used to unambiguously determine peptide secondary structure [38]. We determined the secondary structure of the AzAla-substituted peptides in buffer solution and in the buffer supplemented with phospholipid POPC (1-palmitoyl-2-oleoyl-glycero-3-phosphocholine). All peptides adopted mostly helical structure as expected [29], and interaction with lipid vesicles did not change the structure much (Figure 3).

### 3.5. Fluorescence Studies

The ultimate goal is to demonstrate that AzAla is a suitable substitution for tryptophan in fluorescence experiments; AzAla has a number of spectroscopic properties that make it a unique tool for studies of the mechanism of action of AMPs.

**Figure 4:**
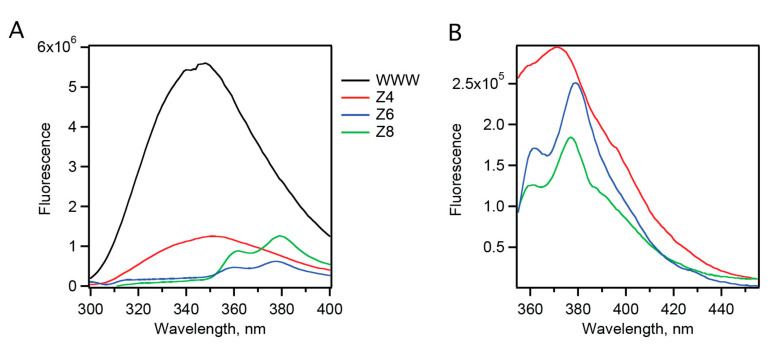
Fluorescence emission spectra of peptides when excited at 280 nm (**A**) and 342 nm (**B**). Conditions: [peptides] = 2 µM, buffer = 50 mM phosphate, 150 mM NaCl, pH 7.0.

Azulene has an absorption spectrum with transitions distinctly different from those of native tryptophan. The AzAla spectrum is dominated by S_1_–S_0_, S_2_–S_0_, and S_3_–S_0_ transitions, centered around 600 nm, 342 nm, and 280 nm, respectively. The S_1_–S_0_ transition has a low extinction coefficient (~400 cm^−1^M^−1^) and does not lead to fluorescence; the S_3_-S_0_ transition overlaps with the Trp absorption spectrum. However, the S_2_–S_0_ transition is both distinct from the Trp absorbance bands and exhibits extinction coefficients and quantum yield similar to Trp in the 280 nm region (Figure S4). The resulting fluorescence emission spectrum of the peptide containing AzAla is compared to the native peptide that has only Trps in Figure 4. Due to the spectral properties, the S_2_ transition of AzAla can be selectively excited in the presence of Trp (Figure 4B). The selective excitation of the AzAla amino acid (λ_ex_ = 342 nm) in the presence of Trp allows for direct interrogation of the single aromatic residue without cross-excitation of the others. This provides for less ambiguity in the analysis of the environment around the AzAla [22,23].

### 3.6. Protease Degradation

**Figure 5:**
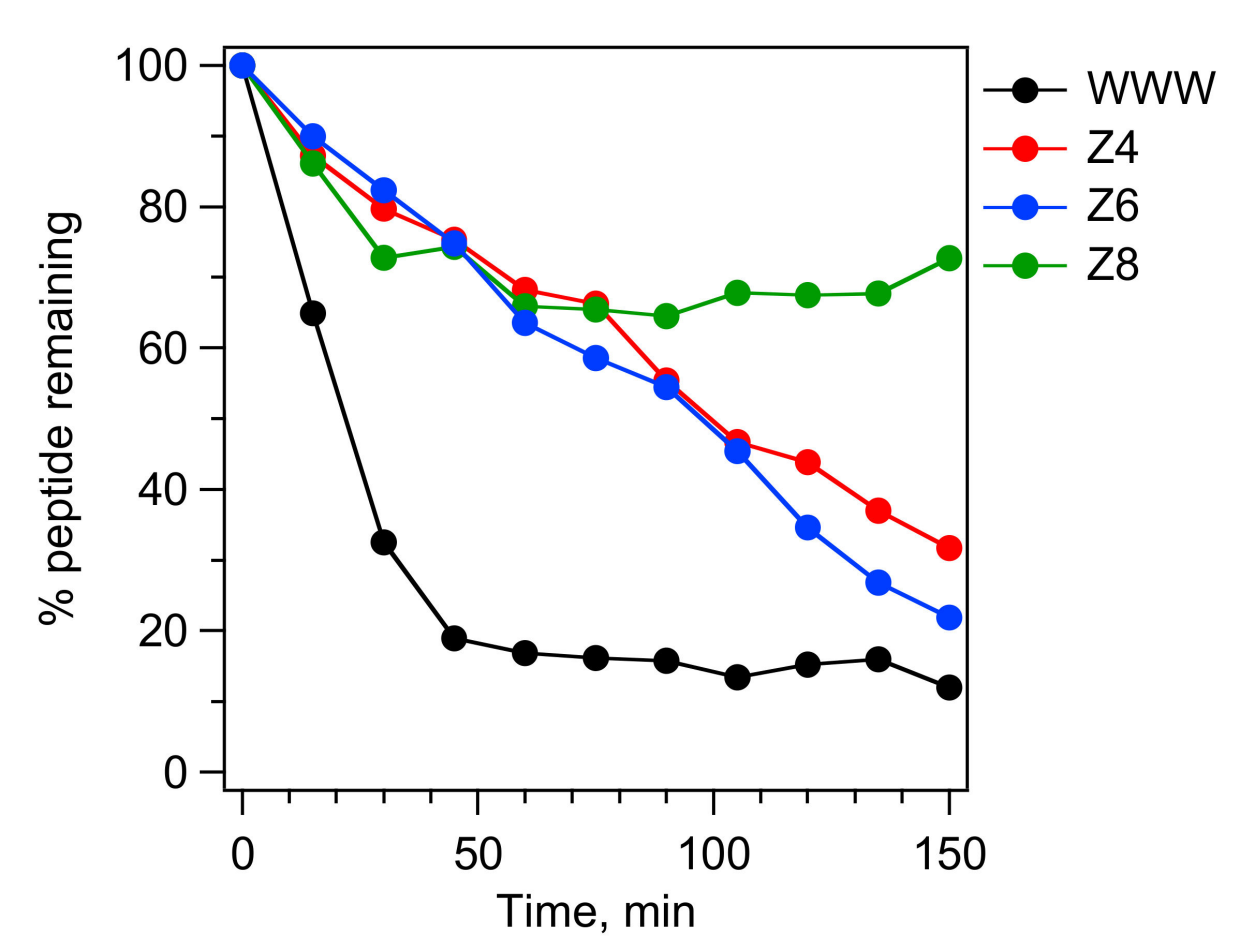
Presence of AzAla in the sequence makes peptides less susceptible to proteolytic degradation. Absorbance of original peptide mixed in a solution with the protease trypsin was monitored over time using analytical HPLC.

For the following experiment we chose trypsin (human endopeptidase that cleaves at arginine or lysine) due to the availability of this protease, although it is mostly found in the digestive system. The goal of this experiment was to provide a proof-of-concept evidence that non-natural amino acids can provide a proteolytic protection. Peptides composed of natural amino acids are susceptible to proteolytic degradation [39]. Proteolytic degradation of peptides is one of the defenses bacteria developed against AMPs; therefore, therapeutic application of peptides and their materials require resistance to proteases [40,41]. In order to gain proteolytic stability, several approaches have been used: (1) changing chirality of peptide from L to D [42,43,44,45,46], (2) mixing L and D peptides [47], (3) use of peptidomimetics [48], and (4) introducing non-natural amino acids [49]. Addition of AzAla to the peptide sequence increases stability of the AMP, with Z8 being the most tolerant to the protease (Figure 5). The mechanism of proteolytic degradation of WWW by trypsin is described in the Figure S5 and Table S1.

### 3.7. Cytocompatibility of AzAla-Containing Peptides

**Figure 6:**
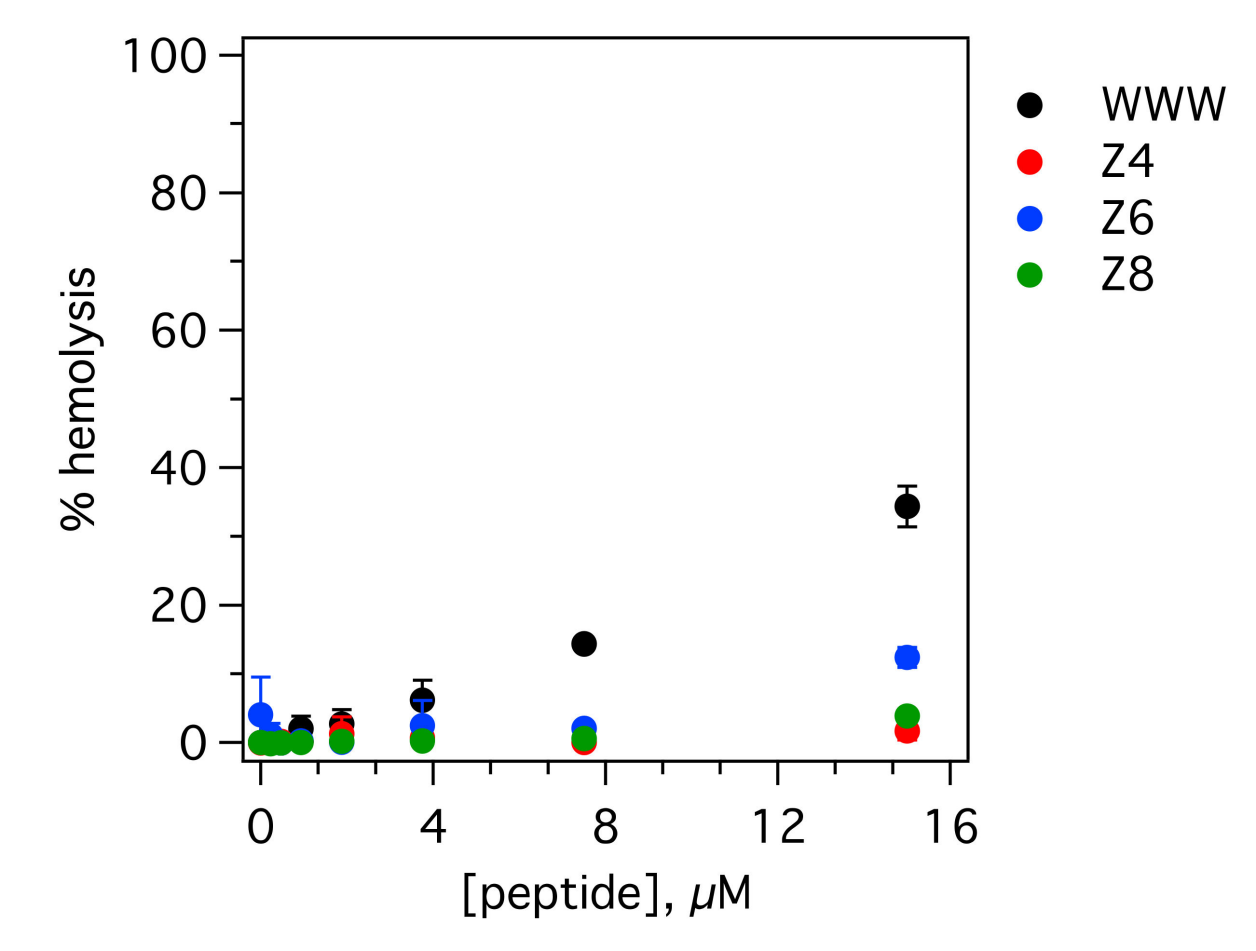
Hemolysis of red blood cells (RBCs) as measured by absorbance at 415 nm.

An important aspect that makes AMPs an attractive target for therapeutic development is the low toxicity these molecules generally exhibit towards host cells. However, with the incorporation of non-natural amino acids, confirming that these modified AMPs retain low levels of cytotoxicity is critical. A widely utilized model system for initial screening for cytotoxicity is a hemolysis assay. The assay relies on the measurement of leakage of hemoglobin from ovine red blood cells (RBCs). Upon incubating the peptides with RBCs, the amount of hemoglobin that has leaked out of the cells is measured using its natural Soret band absorbance at 415 nm. The percent hemolysis exhibited in the samples is calculated by comparing the leakage induced by peptides to that induced by a detergent (positive control) and buffer (negative control). The results are shown in Figure 6. The parent peptide, WWW, induced ~34% hemolysis at the highest concentration tested of 15 µM, and ~15% hemolysis at 7.5 µM which represents the MIC values of *P. aeruginosa* and *E. coli*, respectively. Notably, all of the peptides with the AzAla substitution showed a decrease in hemolysis across all concentrations tested. Specifically, the Z4 and Z8 peptides exhibited a 95% and 89% reduction in hemolysis compared to WWW at 15 µM, respectively. The Z6 peptide exhibited a less dramatic reduction in hemolysis, ~14% at 15 µM representing a 64% reduction. 

**Figure 7:**
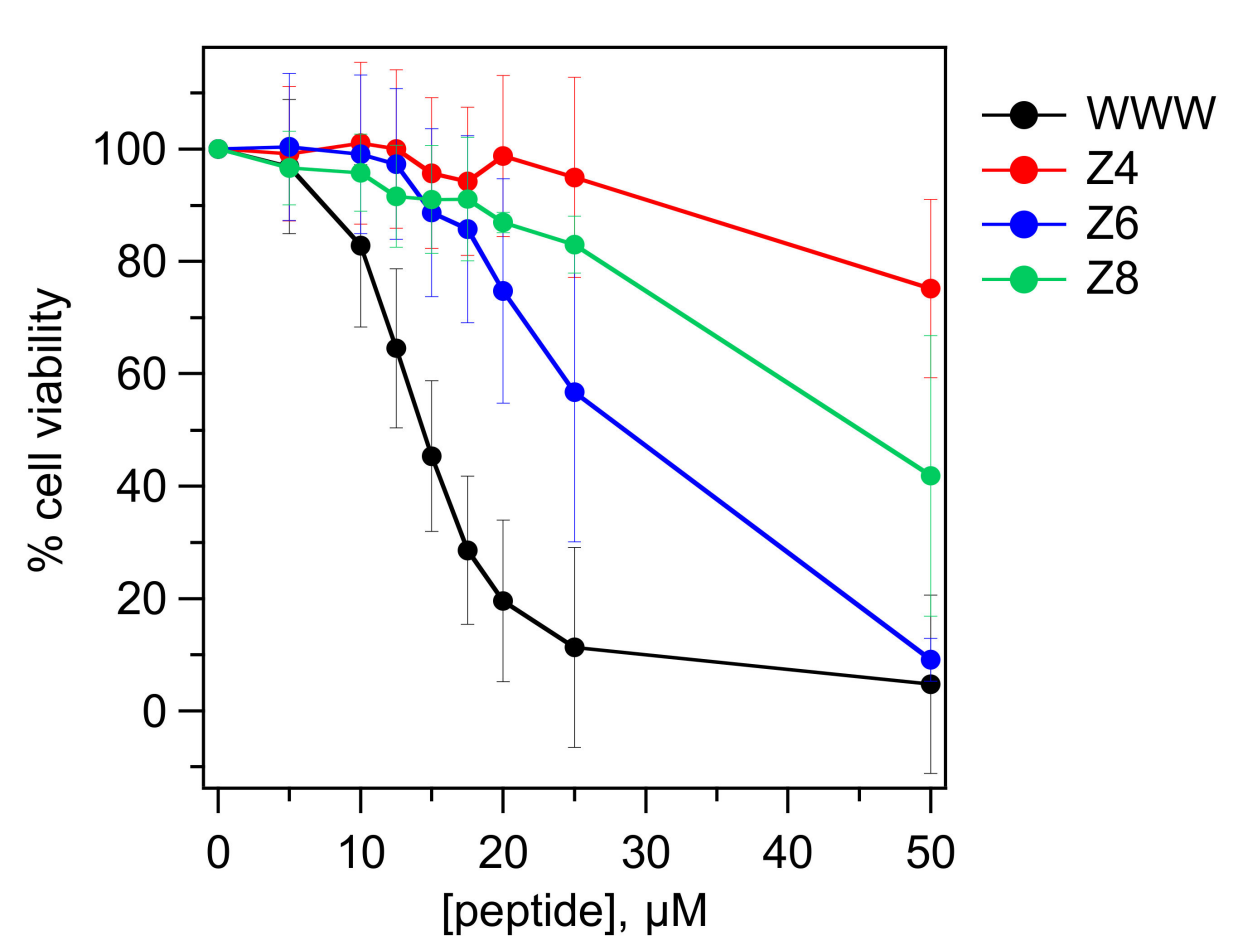
Cytocompatibility of 3T3 mouse fibroblast cells as measured by resazurin assay.

The mammalian cell cytocompatibility of AzAla-substituted peptides was also tested using 3T3 mouse fibroblasts. After cells were incubated with peptides for 5 h, the metabolic activity of cells was assessed using a resazurin-based assay. The amount of living cells is determined based on the fluorescence of resorufin, the product of resazurin reduction by mitochondrial enzymes, which are only active if cells are actively respiring (Figure 7).

## 4. Discussion

In this work, we establish AzAla as a conservative replacement for Trp that changes a peptide’s secondary structure, interaction with lipids, MIC, and even improves the hemolytic and cytocompatibility profiles. The ability to use AzAla in place of Trp is an important tool to deconvolute the contribution of individual tryptophan residues in multi-Trp AMPs to further develop effective antimicrobial agents and a thorough structure-activity relationship in this class of peptides. This approach could also be extended to multi-Trp containing proteins. Additionally, the incorporation of non-natural amino acids improves the resistance of these peptides to proteolytic digestion which may prove to be effective in improving bioavailability.

Naturally occurring AMPs have a wide range of sequences and some have been shown to exhibit dramatic changes in efficacy upon one or more amino acid substitutions. First, we tested the antimicrobial activity of AzAla-substituted peptides against several clinically relevant Gram-negative and Gram-positive strains of bacteria. Antimicrobial efficacy of the wild type peptide and its AzAla-substituted analogs was determined by minimal inhibitory concentration (MIC) and minimal bactericidal concentration (MBC) assays. The data showed that Trp→AzAla substitutions did not impact the MIC and MBC values much when compared to the WT peptide. Notably, the MBC values indicate that the peptides are all acting in a bactericidal manner.

The interaction with a bacterial cell wall is important for the activity of AMPs despite of the mechanism of action. The alteration in amino acid composition can affect the initial interaction and, consequently, the properties of the peptide. The substitution of tryptophan, which is known to interact with the bilayer/headgroup interface, by AzAla can possibly alter the peptide–lipid interactions due to the difference in the side chains. Considering binding to the bacterial membrane is a critical step in antimicrobial activity, we examined if the Trp→AzAla substitution had a negative effect on outer and inner membrane permeabilization using *E. coli* as a model organism. Enzymatic reporter assays utilizing chromophoric substrates showed that substituted peptides induced significantly less outer membrane permeabilization but similar levels of inner membrane permeabilization compared to the WT. This is especially interesting as several groups have reported that Trp-rich AMPs may act through an intracellular mechanism [50,51,52,53]. This is consistent with our results presented here, as the loss of outer membrane permeabilization in the AzAla peptides did not correspond to any changes in the MIC or MBC against *E. coli*.

Structurally, the CD of the free peptides and peptides inserted into model membranes also showed that Trp→AzAla substitution did not alter AMP structure in the buffer and membrane-mimetic membrane. This indicates that the helical propensity of the AzAla-containing peptides is similar to the Trp-containing peptides, which is consistent with previous reports on this amino acid substitution in other peptide backbones [23,24,27]; however, any quantification of the helical propensity would require significant further experimentation. Retention of the helical conformation of the peptide is important when interpreting the proteolytic digestion assays. The data clearly show the substitution provides protection from the proteolytic degradation by trypsin which preferentially cleaves peptide bonds on the C-terminal side of the cationic residues Lys and Arg. As there are no significant changes in the secondary structure of the peptide, these changes in susceptibility are therefore likely caused by the enzyme losing efficiency at recognizing or binding to the peptide with the non-natural amino acid. Further, the Z8 peptide showed the greatest resistance to trypsin, which we speculate is because this residue is the closest to the cationic residues in the peptide which the trypsin recognizes and cleaves. While this hypothesis requires further testing, it provides some guidance as to targeted replacements of natural amino acids with non-natural ones in the design of novel AMPs.

The importance of engineering protease resistance in AMPs is twofold. Numerous proteases exist at the site of an infection. Bacterial proteases promote invasion and delay wound healing [54]. Proteolytic degradation of peptides is one of the defense mechanisms that bacteria developed against antimicrobial peptides; therefore, therapeutic application of peptides require resistance to proteases [40]. Additionally, human matrix metalloproteinases are overproduced in chronic wounds, contributing to delayed would healing [55]. We utilized a commonly available trypsin protease to demonstrate that AzAla can indeed provide resistance to protease degradation. As the initial experiments showed promising results, peptides’ resistance towards bacterial and human proteases will be further investigated. Specifically of interest would be characterization of resistance to proteinase K—a bacterial serine protease that cleaves peptide bonds at the carboxylic sides of aliphatic, aromatic, or hydrophobic amino acids [56], and aureolysin-metalloprotease from *S. aureus* that cleaves at hydrophobic side chains [57,58] and elastase [59]. Beyond infection site proteases, digestion by proteases in the GI tract is a major hurdle to oral bioavailability of AMPs in a therapeutic application [60,61]. Several groups have shown that the incorporation of non-natural amino acids has improved the stability of AMPs to a variety of proteases [2,62,63]. The results shown for all three peptides with a single AzAla substitution are consistent with this trend.

The antimicrobial efficacy of the AzAla-containing AMPs is only valuable if the molecules retain the traditionally low cytotoxicity of naturally occurring AMPs. Cytotoxicity was tested by hemolysis of red blood cells and by fibroblast viability assay. Both approaches demonstrated that AzAla substitution improved the biocompatibility of the AzAla-containing buCATHL-4B analogs with mammalian cells. Hemolysis is often used as an initial estimate of peptide toxicity [64,65], while cytocompatibility assay with fibroblast cells is a more precise method to measure the toxicity of AMPs against skin. The results of both approaches are consistent with the literature data for the wild type (WWW) peptide [29], and AzAla-substituted variants exhibited improved cytocompatibility. While the exact mechanism of cytotoxicity from buCATHL-4B has not been demonstrated, the AzAla substitution clearly counteracts this mechanism.

The combination of increased cytocompatibility and resistance to trypsin are promising early data for the further development of these peptides as antimicrobial therapeutics. By maintaining the antimicrobial efficacy but reducing the cytotoxicity, the AzAla substitution effectively increases the therapeutic window available for these AMPs. Naturally, further investigation in animal models would be necessary to develop a more precise toxicity profile, but the initial results are nonetheless promising.

Beyond the direct case of buCATHL-4B, these results support the application of the AzAla amino acid in the design and development of novel AMPs, as well as in peptide therapeutics with other targets. Specifically, the AzAla could be considered for incorporation into a variety of different Trp-rich AMPs such as Tritpticin, indolicidin, and lactoferricin [50,66]. Other Trp-containing AMPs are equally attractive for exploring these substitutions, such as the gramicidins and the clinically approved Daptomycin, as well as potential substitutions for other aromatic residues in clinically relevant antimicrobials like polymyxin B and Teixobactin, which both contain aromatic groups [60,67,68,69,70]. Further, all peptide therapeutics can be approached from a SAR lens to improve efficacy, bioavailability, and cytocompatibility. Examples of clinically approved peptide therapeutics that contain a Trp residue include Afamelanotide, Semaglutide, and Enfurvitide [71,72]. On a more fundamental level, characterization of the basic biochemical and biophysical properties of non-natural amino acids is essential to designing novel peptides for specific function. Simply put, one of the significant challenges to protein and peptide design using non-natural amino acids is the lack of detailed information on amino acid characteristics in a wide range of sequences or environments. Through careful and systematic characterization, the application of non-natural amino acids in protein and peptide design can be greatly enhanced.

## 5. Conclusions

In summary, we investigated if non-natural amino acid AzAla could be used to replace tryptophan in one AMP sequence that naturally contains three tryptophan side chains. Fluorescence studies of AzAla-substituted peptides showed that AzAla could be excited separately from the remaining present tryptophan residues. CD experiments confirmed that the structure of the peptide does not change much as compared to the original peptide in the buffer and also in lipid vesicles; therefore, AzAla is a suitable replacement for the tryptophan in AMPs and could be used to study the mechanism of AMP function. In addition, we found that the substitution confers a number of benefits onto the original peptide. AzAla-substituted peptides have lower hemolysis and higher cytocompatibility as compared to the WT peptide, while preserving original antimicrobial efficacy. In addition, we demonstrated that AzAla provides protection against proteases, such as trypsin. Currently, we are investigating if AzAla-containing peptides also survive longer in the presence of naturally occurring proteases on the mammalian skin surface. Taken together, our work concludes that AzAla not only preserves natural features of antimicrobial peptides, but also provides beneficial properties. Therefore, AzAla as a Trp analog could be used in other multi-Trp AMPs, such as porcine host defense peptides Tritrpticin [73] or Lys-C (fragment of hen egg white lysozyme [74]). The machinery to incorporate AzAla into bigger proteins is available [75]. Given the rise in antibiotic resistance [76], these findings might result in more efficient AMPs for biomedical applications.

Overall, the AzAla amino acid has the potential to become a useful tool in the design and development of novel, functional peptides. The unique spectral properties coupled with the apparent beneficial impacts on cytocompatibility, and protease resistance can yield application to a number of fundamental and therapeutic systems.

## Figures and Tables

**Table 1:** Sequences of buCATHL-4B AzAla-derivatives used in this study (Z denotes AzAla).

Peptide	Sequence	MW (Calculated)
WWW	AIPWIWIWRLLRKG	1806.1
Z4	AIP**Z**IWIWRLLRKG	1817.0
Z6	AIPWI**Z**IWRLLRKG	1817.0
Z8	AIPWIWI**Z**RLLRKG	1817.0

**Table 2:** Bacterial growth inhibition/killing concentration (µM).

	*E. coli*		*S. aureus*		*P. aeruginosa*		*A. baumannii*	
	MIC	MBC	MIC	MBC	MIC	MBC	MIC	MBC
Z4	7.50	7.50	7.50	7.50	>15	>15	7.50	7.50
Z6	7.50	7.50	1.88	1.88	>15	>15	3.75	7.50
Z8	7.50	7.50	7.50	15.00	>15	>15	7.50	7.50
WWW ^a^	7.50	7.50	3.75	3.75	15.00	>15	3.75	3.75

^a^ Data from ref. [30].

## Data Availability

Not applicatble.

## Supplementary Materials

The following are available online at https://www.mdpi.com/2218-273X/11/3/421/s1, Figure S1: Minimal Bactericidal Concentration (MBC). MBC was carried out by transferring 1 μL from each well of the MIC experimental 96-well plates onto antibiotic-free LB agar and allowed to grow overnight at 37 °C. Plates were photographed the next morning and MBC was determined visually by the presence/lack of colony growth., Figure S2: Time course of E. coli outer membrane leakage. Ezymatic hydrolysis of nitrocefin by β-lactamase was determined by increases in absorbance at 486 nm. The samples were monitored in 5-min intervals for a total of 90 min. Samples contained varying amounts of serially diluted peptides (A) Z4, (B) Z6, (C) Z8, or (D) Polymyxin-B sulfate. Peptide concentrations shown are in micromolar units. Data shown are the averages of 3 trials with standard deviations (in some cases smaller than the symbol size)., Figure S3: Time course of *E. coli* inner membrane leakage. Ezymatic hydrolysis of ONPG by β-galactosidase was determined by increases in absorbance at 420 nm. The samples were monitored in 5-minute intervals for a total of 90 min. Samples contained varying amounts of serially diluted peptides (A) Z4, (B) Z6, (C) Z8, or (D) CTAB. Peptide concentrations shown are in micromolar while CTAB concentrations are in millimolar units. Data shown are the averages of 3 trials with standard deviations (in some cases smaller than the symbol size)., Figure S4. Absorption spectra of AzAla-substituted peptides., Figure S5. Trypsin digest of WWW peptide (AIPWIWIWRLLRKG) (A). Overlay of chromatograms acquired at various time points after peptide and trypsin were mixed. The inset graph shows the percentage of undigested peptide peak area monitored over time. Identity of peaks was established by MALDI-TOF using the sample that was digested for 4 hours (see Table S1 below). (B) MALDI-TOF analysis of peak with retention time of 8.3 min: m/z peak at 1237 Da was assigned to AIPWIWIWR peptide segment. (C) MALDI-TOF analysis of peak with retention time of 8.59 min. The peak at 1802 Da was assigned to the undigested peptide. (D) MALDI-TOF analysis of the peak with retention time of 8.87 min. The peak at 1618 Da corresponds to AIPWIWIWRLLR peptide segment. Table S1. Fragments of peptides observed by MALDI-TOF after trypsin digestion. Individual fragments were identified by collecting various peaks after HPLC separation.
