# Supplementary material for: Beneficial Impacts of Incorporating the Non-Natural Amino Acid Azulenyl-Alanine into the Trp-Rich Antimicrobial Peptide buCATHL4B
Source: Biomolecules. 2021 Mar 12;11(3):421. doi: 10.3390/biom11030421 (PMC8001250; doi:10.3390/biom11030421)
Supplement: Supplementary file 1 [file biomolecules-11-00421-s001.pdf]

Beneficial Impacts of Incorporating the Non-Natural Amino Acid Azulenyl-Alanine into the Trp-Rich Antimicrobial Peptide buCATHL4B

Areetha R. D'Souza [1], Matthew R. Necelis [2], Alona Kulesha,[1] Gregory A. Caputo [2,3], and Olga V. Makhlynets [1]*

[1] Department of Chemistry, Syracuse University, 111 College Place, Syracuse, NY
[2] Department of Chemistry & Biochemistry, Rowan University, Glassboro NJ USA
[3] Department of Molecular & Cellular Biosciences, Rowan University, Glassboro NJ USA

SUPPORTING INFORMATION

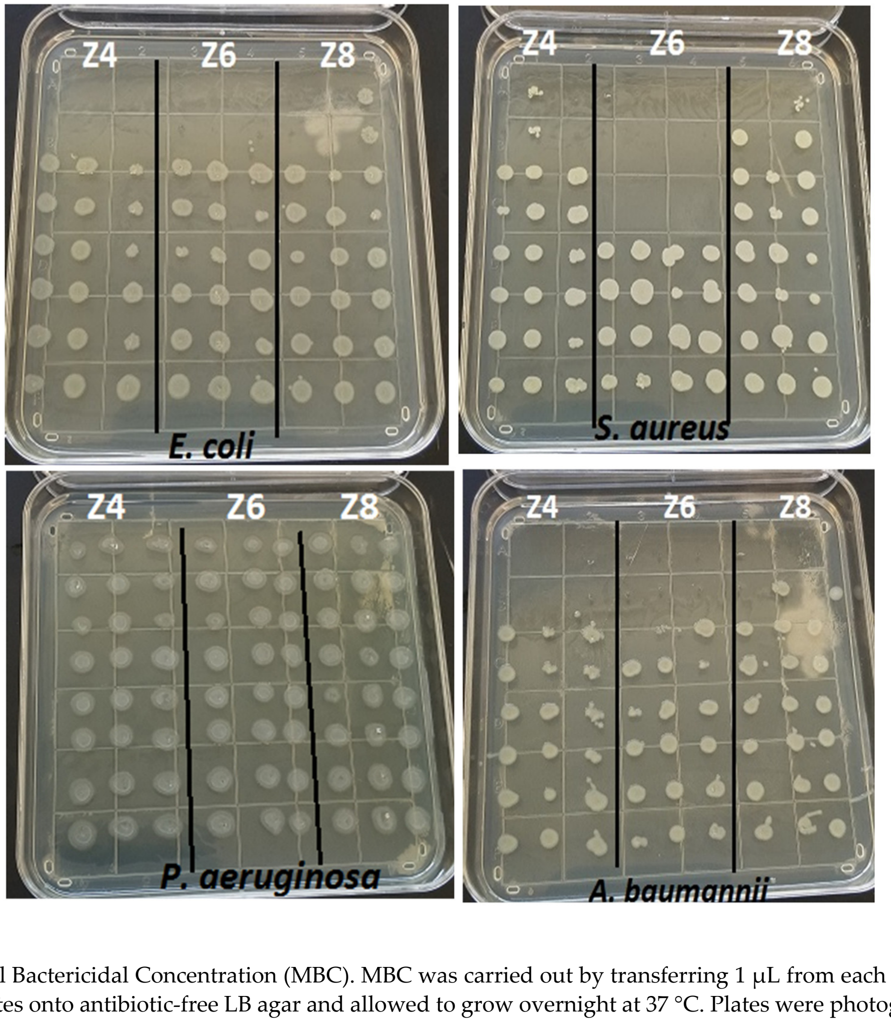

Figure S1. Minimal Bactericidal Concentration (MBC). MBC was carried out by transferring 1 μL from each well of the MIC experimental 96-well plates onto antibiotic-free LB agar and allowed to grow overnight at 37 °C. Plates were photographed the next morning and MBC was determined visually by the presence/lack of colony growth.

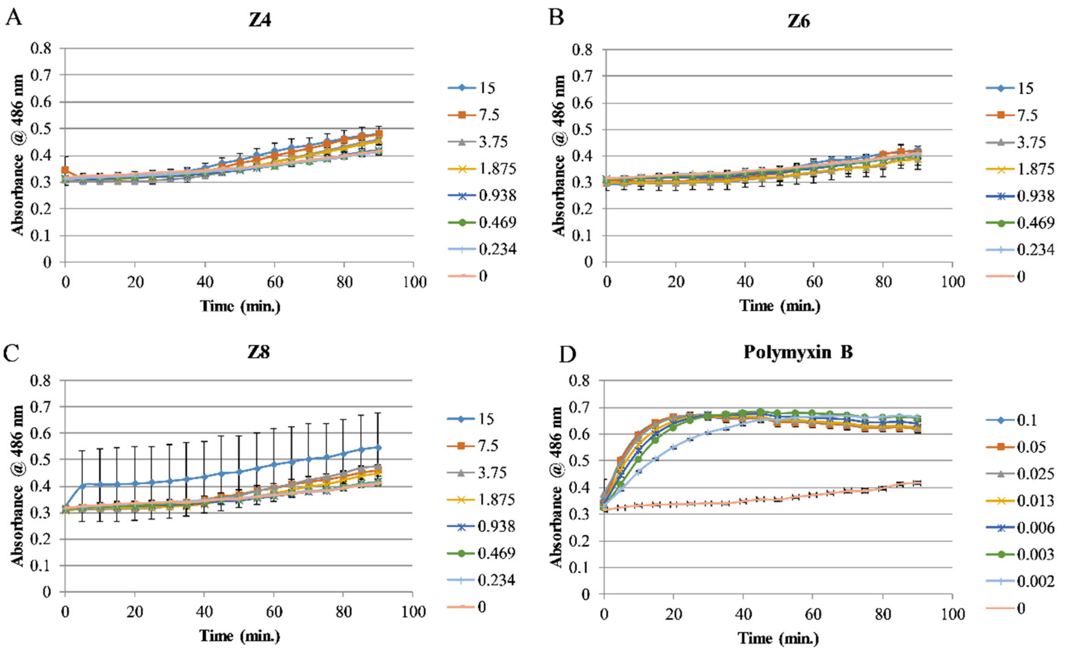

Figure S2. Time course of *E. coli* outer membrane leakage. Ezymatic hydrolysis of nitrocefin by β-lactamase was determined by increases in absorbance at 486 nm. The samples were monitored in 5-minute intervals for a total of 90 minutes. Samples contained varying amounts of serially diluted peptides (**A**) Z4, (**B**) Z6, (**C**) Z8, or (**D**) Polymyxin-B sulfate. Peptide concentrations shown are in micromolar units. Data shown are the averages of 3 trials with standard deviations (in some cases smaller than the symbol size).

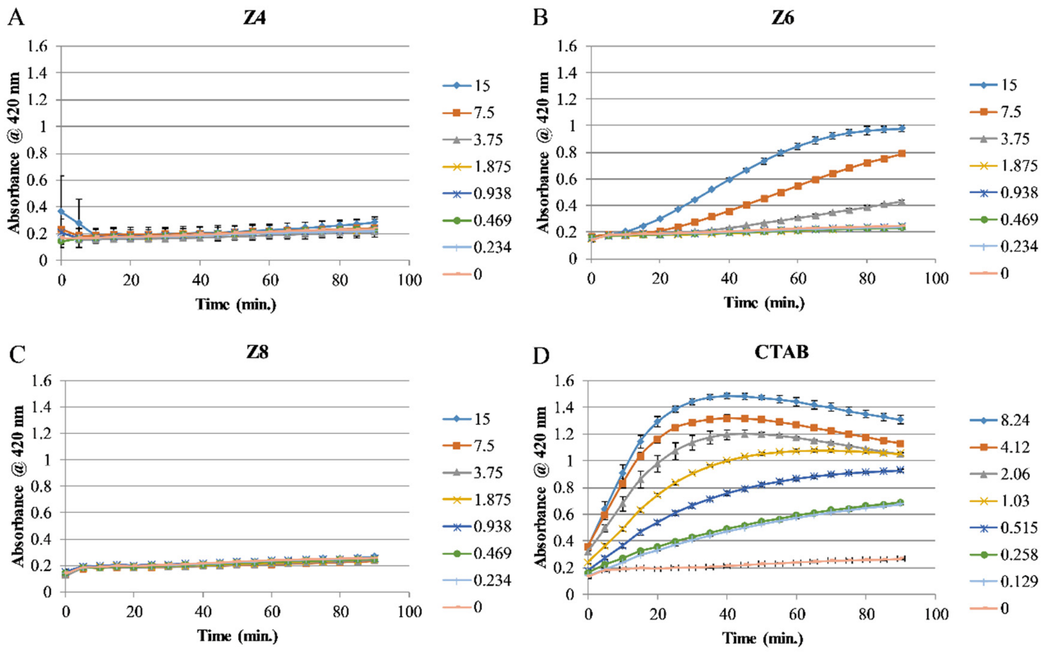

Figure S3. Time course of *E. coli* inner membrane leakage. Ezymatic hydrolysis of ONPG by β-galactosidase was determined by increases in absorbance at 420 nm. The samples were monitored in 5-minute intervals for a total of 90 minutes. Samples contained varying amounts of serially diluted peptides (**A**) Z4, (**B**) Z6, (**C**) Z8, or (**D**) CTAB. Peptide concentrations shown are in micromolar while CTAB concentrations are in millimolar units. Data shown are the averages of 3 trials with standard deviations (in some cases smaller than the symbol size).

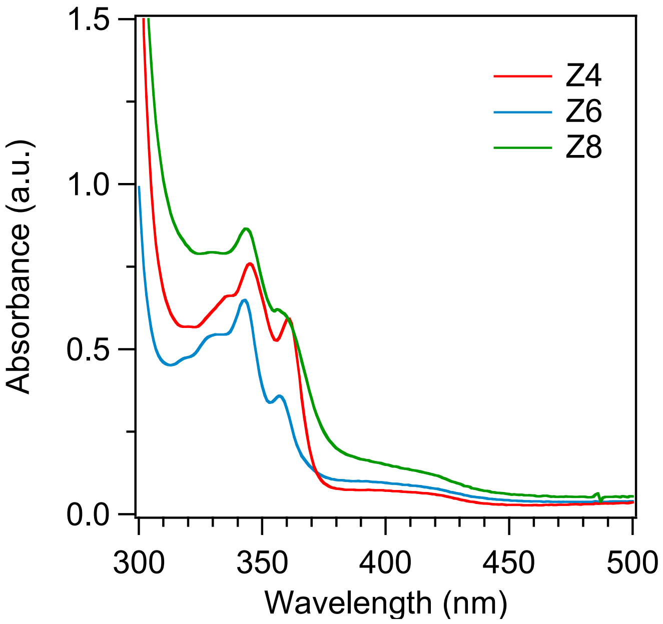

Figure S4. Absorption spectra of AzAla-substituted peptides.

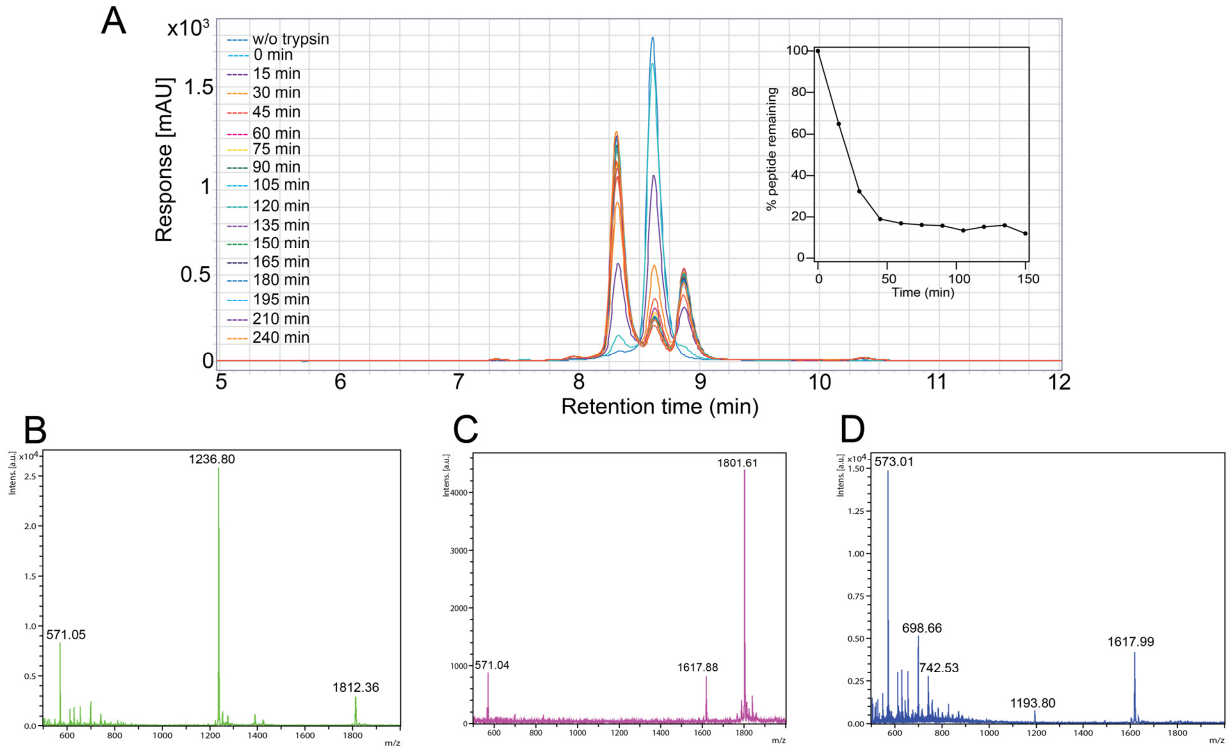

Figure S5. Trypsin digest of WWW peptide (AIPWIWIWRLLRKG) (**A**). Overlay of chromatograms acquired at various time points after peptide and trypsin were mixed. The inset graph shows the percentage of undigested peptide peak area monitored over time. Identity of peaks was established by MALDI-TOF using the sample that was digested for 4 hours (see Table S1 below). (**B**) MALDI-TOF analysis of peak with retention time of 8.3 min: m/z peak at 1237 Da was assigned to AIPWIWIWR peptide segment. (**C**) MALDI-TOF analysis of peak with retention time of 8.59 min. The peak at 1802 Da was assigned to the undigested peptide. (**D**) MALDI-TOF analysis of the peak with retention time of 8.87 min. The peak at 1618 Da corresponds to AIPWIWIWRLLR peptide segment.

Table S1. Fragments of peptides observed by MALDI-TOF after trypsin digestion. Individual fragments were identified by collecting various peaks after HPLC separation.

**Peptide Sequence**	**Position of the Peak in the Chromatogram**	**Maldi-Tof Signal, DA**	**Expected Mass, DA**
Aipwiwiwr	1	1237	1240
Aipwiwiwrllrkg *	2	1802	1806
Aipwiwiwrllr	3	1618	1622

* amide at C-term.
